# Viability-PCR for the selective detection of *Lactobacillus acidophilus* and *Bifidobacterium bifidum* in live bacteria-containing products

**DOI:** 10.3389/fmicb.2024.1400529

**Published:** 2024-07-03

**Authors:** Stefania Catone, Serena Iannantuono, Domenico Genovese, Christina Von Hunolstein, Giovanna Franciosa

**Affiliations:** Biologicals and Biotechnologicals Unit, Istituto Superiore di Sanità, National Center for the Evaluation and Control of Medicines, Rome, Italy

**Keywords:** viability-PCR, plate count enumeration, qPCR, *Lactobacillus acidophilus*, *Bifidobacterium bifidum*, live bacteria-containing products

## Abstract

To exert their beneficial effects, microorganisms used in live bacteria-containing products must be viable and present in certain amounts. In this study, we developed a viability assay based on quantitative PCR coupled with propidium monoazide for the identification and enumeration of viable *Lactobacillus acidophilus* and *Bifidobacterium bifidum*. In order to optimize the protocol, the thermal inactivation conditions for the two target microorganisms and the PMA concentration inhibiting DNA amplification from the dead cells while allowing it from the live cells were first determined. The viability-PCR protocol was then applied to analyze a commercial product containing the two microorganisms. The quantities of both microorganisms determined using viability-PCR in the tested product were significantly higher than those obtained using the standard plate count, suggesting the presence of bacteria in a viable but non-culturable physiological state. Moreover, lower amounts of the two microorganisms were detected using viability-PCR compared to those achieved using quantitative PCR, possibly because of the presence of dead cells in the samples. Our results suggest that the viability-PCR method proposed here is a suitable alternative for rapid and accurate quantification and assessment of the viability of *L. acidophilus* and *B. bifidum* and could be easily adopted in the quality control screening of live bacteria-containing products.

## 1 Introduction

The qualitative and quantitative compositions of the human gut microbiota change in health and disease status. Therefore, maintaining a balanced gut microbiota or restoring it from perturbation may significantly maintain and improve health (Laudes et al., 2021; Afzaal et al., 2022).

Microorganisms in spontaneously fermented foods have been empirically used for these purposes (Leeuwendaal et al., 2022). Subsequent studies have revealed that only certain live microbial strains in abundant quantities can confer health benefits to the host, mainly by enhancing metabolic functions, strengthening the mucosal intestinal barrier, protecting against pathogens, and stimulating the immune system (Campaniello et al., 2023; Skoufou et al., 2024). Scientific advances in the field have promoted the expansion and diversification of products containing live bacteria, including probiotic food supplements commonly used to ameliorate intestinal and general health, live biotherapeutic products intended to prevent or treat several diseases, and fecal microbiota that, once transferred from healthy donors to individuals with intestinal disorders, can restore the gut microbial balance (Mcilroy et al., 2019; Cordaillat-Simmons et al., 2022; Franciosa et al., 2023).

While the fecal microbiota consists of undefined microbial communities (Kump et al., 2018), both probiotic products and biotherapeutics (hereinafter collectively referred to as live bacteria-containing products, LBCP) include single or multiple microorganisms that must unequivocally be identified, viable, and administered in adequately high numbers to be effective (Hill et al., 2014; Campaniello et al., 2023). Therefore, for the manufacturing and regulation of LBCP, the three quality criteria—identification, viability, and quantity—should be reported in their labels and fulfilled throughout the product shelf life (FAO/WHO, 2002; Council for Responsible Nutrition International Probiotics Association, 2017; European Pharmacopoeia Commission, 2019).

Culture-dependent methods are typically applied for monitoring the production of LBCP and verifying label compliance. However, culture techniques have disadvantages that affect each of the above specified criteria requirements: (i) microbial identification may be challenging when multiple strains are used in the same product, especially if they have similar physiological properties and growth requirements; (ii) microbial viability may not always be detected by culturing, as some microbial cells may enter a viable but non-culturable (VBNC) physiological status in response to environmental stresses, losing cultivability while retaining metabolic activity and membrane integrity; and (iii) culture-based microbial quantification presents high coefficients of variation (Davis, 2014; Bagheripoor-Fallah et al., 2015; Boyte et al., 2023).

Alternative methods have been developed for testing LBCP, such as flow cytometry, mass spectrometry, or molecular approaches including whole-genome and next-generation sequencing; however, most fail to concomitantly provide the identification and absolute quantification of viable microorganisms (Angelakis et al., 2011; Pane et al., 2018; Sharma et al., 2020; Zawistowska-Rojek et al., 2022).

Quantitative PCR (qPCR) in combination with propidium monoazide (PMA), a method also referred to as viability-PCR (vPCR), has been used to detect and enumerate viable microorganisms, including lactic acid bacteria, in different matrices (García-Cayuela et al., 2009; Shao et al., 2016; Lai et al., 2017; Scariot et al., 2018; Shehata and Newmaster, 2021; Shi et al., 2022; Shehata et al., 2023; Marole et al., 2024). PMA can distinguish between live and dead cells as it enters dead bacteria with damaged membranes while being excluded from intact living bacteria; once in the compromised cells, PMA covalently binds the genomic DNA in the presence of strong visible light, thus preventing subsequent DNA amplification from dead bacteria and eliminating overestimation of the bacterial counts in qPCR assays (Nocker et al., 2007).

Here, we developed a vPCR protocol for rapid and accurate identification and enumeration of viable *Lactobacillus acidophilus* and *Bifidobacterium bifidum*, two microbial species frequently used in LBCPs. After optimization, the vPCR protocol was verified using different mixtures of live and dead cells obtained using thermal inactivation. Finally, a commercial LBCP was analyzed using vPCR, and the results were compared with those obtained using traditional plate count and qPCR.

The goal of the study was to provide the regulatory bodies and the manufacturers with a rapid and high-throughput method for the microbiological quality assessment of LBCPs.

## 2 Materials and methods

### 2.1 Bacterial strains and growth conditions

All strains were purchased from the German Collection of Microorganisms and Cell Cultures (DSMZ), except for *Enterococcus faecium* SF68 which was from the Istituto Superiore di Sanità culture collection. The strains were stored at −80°C in cryogenic vials (Prolab Diagnostics).

*L. acidophilus* (DSM 20079) and *B. bifidum* (DSM 20456) were used as reference strains for vPCR. *L. delbrueckii* subsp. *bulgaricus* (DSM 20081); *L. delbrueckii* subsp. *lactis* (DSM 20072); *Lactiplantibacillus plantarum* (DSM 20174); *Lacticaseibacillus paracasei* (DSM 5622); *B. animalis* subsp. *lactis* (DSM 10140); *B. breve* (DSM 20213); *Bacillus clausii* (DSM 8716); and *Streptococcus thermophilus* (DSM 20617), which are the most frequently found in LBCPs available in Italy, and *E. faecium* SF68 were used to confirm the specificity of the primers/probe sets used in qPCR and vPCR.

Bacteria were grown in de Man, Rogosa, Sharpe (MRS) broth or agar (Oxoid), supplemented with 0.05% L-cysteine HCl for culturing the more strictly anaerobic bifidobacteria strains, and incubated at 37°C under aerobic or anaerobic conditions, depending on the bacterial species requirements. Anaerobic jars and gas generating kits (Oxoid) were used to simulate the anaerobic conditions.

### 2.2 Enumeration of viable *L. acidophilus* and *B. bifidum* using standard plate count

Plate count (PC) enumeration of *L. acidophilus* and *B. bifidum* in pure broth cultures was performed as previously described (Aureli et al., 2008). Briefly, the test samples were 10-fold diluted in 0.9% saline, and 100 μl of three consecutive dilutions were spread in duplicates on MRS agar or MRS agar supplemented with 0.05% L-cysteine HCl for enumerating *L. acidophilus* and *B. bifidum*, respectively. The plates were then incubated at 37°C for 48–72 h under anaerobic conditions.

Subsequently, the total number of *L. acidophilus* or *B. bifidum* was enumerated in plates containing 30–300 presumptive colonies, and counts were recorded as colony-forming units (CFU) per milliliter of broth culture. All experiments were repeated three times for each reference microorganism, and the results expressed as mean ± standard deviation.

### 2.3 qPCR reactions and conditions

Genomic DNA was isolated from pure microbial broth cultures grown overnight using the Qiagen DNEasy Blood and Tissue kit, following the manufacturer's instructions. The quality and quantity of isolated DNA were estimated using an ultraviolet spectrophotometer (Biophotometer, Eppendorf). The DNA samples were stored at −20°C until use.

The two primer-probe sets used in separate qPCR reactions to detect *L. acidophilus* and *B. bifidum* were selected from the literature (Haarman and Knol, 2006; Singh et al., 2013) and their nucleotide sequences are reported in Table 1. The probes were labeled with the reporter molecule 6-carboxyfluorescein (FAM) and quencher tetramethylrhodamine (TAMRA) at the 5′-end and 3′-end respectively.

**Table 1:** Oligonucleotide primers ad probes used in this study.

**Microbial species**	**Primer or probe name**	**Sequence (5^′^–3^′^)**	**Target region**	**Reference**
*L. acidophilus*	F_acid R_acid Probe_acid	GAAAGAGCCCAAACCAAGTGATTCTTCCCAGATAA TTCAACTATCGCTTATACCACTTTGCAGTCCTACA	16S-23S intergenic spacer region	Haarman and Knol (2006)
*B. bifidum*	F_bifid R_bifid Probe_bifid	ACCGAATTCGCCTGTCACTTACGGCGCGGATTCGT CCGCTGGATGTGAAC	*oppD* gene^*^	Singh et al. (2013)

^*^Oligopeptide transport ATP-binding protein.

The qPCR reaction mixtures (20 μl final volume) were prepared in duplicates and consisted of 10 μl of 2X TaqPath qPCR Master Mix (Applied Biosystems), 1 μl of 20X primer-probe set (Integrated DNA Technologies, IDT), 5 μL of template DNA, and 4 μl of DNAse/RNAse-free water (Bioline). Two control replicates without a DNA template were included in each run.

Real time qPCR amplification was performed in a MicroAmp optical 96-well reaction plates sealed with optical adhesive covers (Applied Biosystems), using a 7,500 Real-Time PCR system (Applied Biosystems). The thermal cycling conditions included pre-incubation at 50°C for 2 min, an incubation step at 95°C for 10 min to activate the AmpliTaq Gold polymerase, 45 cycles at 95°C for 15 s and 60°C for 30 s, and a final incubation step at 60°C for 1 min. The fluorescence signal was measured at the end of each 60°C step. The threshold cycle (Ct) value, corresponding to the PCR cycle number at which fluorescence was detected above the threshold, was calculated using the 7,500 System software (Applied Biosystems). All the above assays were performed twice.

The specificity of the two primer-probe sets for *L. acidophilus* and *B. bifidum* was tested using genomic DNA isolated from all the strains described above.

To determine the absolute quantities of *L. acidophilus* and *B. bifidum* in unknown samples, the Ct value of each sample was compared to the corresponding standard curves, which were constructed using 10-fold serial dilutions of genomic DNA at known concentrations, isolated from the reference strains *L. acidophilus* DSM 20,079 and *B. bifidum* DSM 20,456. The number of microorganisms in the original broth cultures was determined using the PC method and expressed as CFU/ml. The DNA dilutions used for the standard curves were selected to represent at least five bacterial concentrations, ranging from 10^2^ to 10^7^ CFU/ml. The DNA extracts were aliquoted undiluted and stored at −20°C before subsequent single use for standard curve construction.

### 2.4 vPCR set up

#### 2.4.1 Determination of the thermal inactivation conditions for L. acidophilus and B. bifidum

Bacterial pellets from overnight broth cultures of the reference strains *L. acidophilus* DSM 20,079 and *B. bifidum* DSM 20,456 were collected via centrifugation, washed with 0.9% NaCl, and resuspended in the same saline solution to achieve a density at 600 nm (OD600) of ~ 1. Viable cell concentrations in the bacterial suspensions were determined using PC, as described above. Each strain suspension was then subjected to the following thermal inactivation treatments: 75°C for 30 min, 80°C for 20 min, 90°C for 15 min, or 100°C for 10 min. Lethality was verified by culturing on MRS agar plates. The untreated controls for each strain were included in the experiments. To test whether heating caused any DNA modification that affected the qPCR results, total DNA was isolated from both thermally-treated and -untreated samples using a Mag-Bind cfDNA kit (Omega Bio-Tek) and subjected to qPCR, as described above.

#### 2.4.2 Optimization of the PMA concentration for sample pretreatment before qPCR

Reference strain suspensions with OD600 ~ 1 were prepared as previously described, and cell concentrations were determined using PC. Each suspension was then split into two equal volumes, one was left untreated (live cells) and the other thermally inactivated at the conditions defined in the previous experiment to obtain dead cells. The absence of viable cells in the heat-treated samples was verified using PC.

Prior to use, PMA 20 mM (Biotium) was diluted to 2.5 mM with sterile water and stored on ice in the dark. The diluted PMA was then added to duplicate aliquots (250 μl) of live and dead cells, to achieve the final concentrations of 25 μM, 50 μM, and 100 μM. The resulting suspensions were incubated in the darkness for 10 min under gentle agitation. Aliquots of live and dead cells that were not mixed with PMA were used as controls.

All samples, with and without PMA, were placed on ice and photoactivated for 5 min using a 500 W halogen light source located at 20 cm distance from the samples.

After photoactivation, DNA was isolated from the samples using a Mag-Bind cfDNA kit and subjected to qPCR (two replicates per sample).

Moreover, to assess PMA cytotoxicity, bacterial counts in PMA-treated live cell samples were determined using PC and compared with those in PMA-untreated live cell samples.

The assays were repeated four times for each reference microorganism.

### 2.5 Verification of optimal PMA pretreatment for distinguishing between live and dead microbial cells

For each reference strain, live and dead cells in known amounts were prepared as described above and subjected to qPCR, either separately or in different combinations, with or without PMA pretreatment.

Regarding the preparation of bacterial mixtures, aliquots (250 μl) of live cells from broth cultures at OD600 ~ 1 (concentrations of ~10^8^ CFU/ml and ~10^7^ CFU/ml for *L. acidophilus* and *B. bifidum*, respectively) were placed in 1.5 ml tubes. Subsequently, equal volumes of dead cells, which were thermally treated under optimal conditions as described above to ensure zero viability, were added to each 1.5 ml tube containing the live cells at decreasing concentrations (i.e., 10^8^, 10^6^, and 10^4^ CFU/ml for *L. acidophilus* and 10^7^, 10^5^, and 10^3^ CFU/ml for *B. bifidum*).

A complementary experiment was performed using the same approach, except that decreasing concentrations of live cells were added at fixed amounts of dead cells.

Individual dead and live cells were used as controls. The cell mixtures and individual cells were treated then with 25 μM PMA as previously described. Two replicates were used for each cell mixture and control.

Finally, DNA was isolated from all samples using a Mag-Bind cfDNA kit and subjected to qPCR. All the experiments were repeated twice for each reference microorganism.

### 2.6 Identification and quantification of *L. acidophilus* and *B. bifidum* in a commercial product using PC, qPCR, and vPCR

Commercial LBCP capsules containing at least 10^9^ cells of both *L. acidophilus* and *B. bifidum* per capsule, according to the product label, was purchased from the market, stored at 4°C, and analyzed within the expiration date. Five LBCP capsules were analyzed. Before analysis, each LBCP capsule was dissolved in 10 ml of a 0.9% NaCl solution.

PC enumeration of *L. acidophilus* and *B. bifidum* was performed as described previously. The two microorganisms were differentiated based on colony morphology on MRS agar plates and representative colonies were confirmed at the species level using 16SrRNA sequencing, as previously described (Boye et al., 1999). For *B. bifidum*, selective counting was also performed on Bifidum Selective Medium (BSM) agar plates (Millipore).

For vPCR, 1 ml of the capsule suspensions were mixed with 25 μM PMA and photoactivated at the conditions above described. A DNeasy kit was used to isolate DNA from 1 ml of PMA-untreated and -treated capsule suspensions. DNA samples were 100-fold diluted and subjected to qPCR using *L. acidophilus* and *B. bifidum* specific primer/probe sets in separate reactions. Negative controls without templates were included in each run. Each reaction was performed in duplicate. Serial dilutions of the DNA standards were performed in duplicate for each qPCR run. Concentrations of the individual species were plotted against the corresponding standard curve, with the slope and linear correlation of the curves automatically calculated using the AB 7,500 system software.

### 2.7 Statistical analyses

Statistical analysis was performed using the GraphPad Prism 10 software (GraphPad Software). Student's *t*-test and analysis of variance (ANOVA) were used to compare treatment pairs. Differences between treatments were considered statistically significant at *p* < 0.05.

## 3 Results

### 3.1 Specificity of primers and probes, and qPCR standard curves

The specificity of each primer/probe set used in this study has already been assayed, with positive qPCR reactions using *L. acidophilus* and *B. bifidum*, and no cross-reactions reported using several non-target bacteria (Haarman and Knol, 2006; Singh et al., 2013). Here, we confirmed the specificity of the primer/probe sets by testing 11 non-target microorganisms other than those previously evaluated (data not shown).

The standard curves of each microorganism showed a strong linear correlation (r^2^ = 0.9983 and r^2^ = 0.9770 for *L. acidophilus* and *B. bifidum*, respectively) between the Ct values and cell counts in the tested range (10^2^-10^7^ CFU/ml) (95% confidence interval), confirming the high accuracy of the qPCR assays. The amplification efficiencies (E) calculated using the formula E = 10^(−1/slope)^ – 1 (Rasmussen, 2001) were 98% for *L. acidophilus* and 97% for *B. bifidum*.

### 3.2 Thermal inactivation conditions for *L. acidophilus* and *B. bifidum*

Two of the applied thermal treatments (i.e., at 90°C for 15 min and 100°C for 10 min) efficiently inactivated both *L. acidophilus* and *B. bifidum*, as confirmed by the absence of bacterial growth on MRS agar plates following treatments. Although exposures to 75°C and 80°C for 25 min and 20 min, respectively, were also lethal for *B. bifidum*, they did not ensure 100% mortality of *L. acidophilus*, as indicated by the growth of a few colonies on solid media.

For both microorganisms, none of the heat treatments modified the qPCR Ct values compared with the corresponding untreated samples (data not shown).

Based on the above results, heat treatment at 100°C for 10 min was selected as the ideal thermal inactivation treatment to ensure zero viability of both target bacteria.

### 3.3 Optimization of PMA concentration

An optimal PMA concentration should allow the exclusive detection of viable microbial cells, while causing the minimal cytotoxic effects.

**Figure 1:**
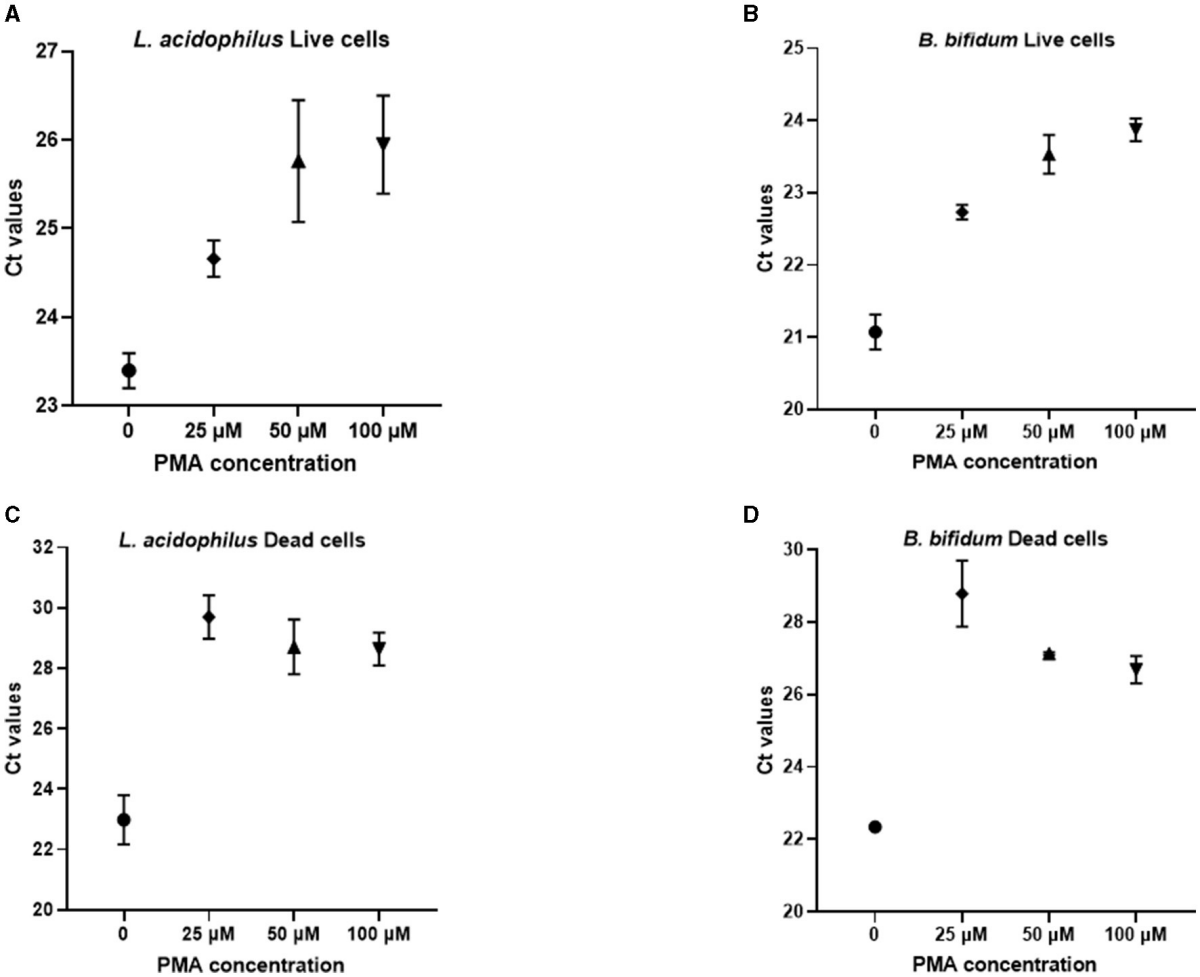
Ct values obtained from qPCR experiments after pretreatment of *L. acidophilus* and *B. bifidum* live cells **(A, B)** and dead cells **(C, D)** with different PMA concentrations.

For selecting the PMA concentration that adequately distinguished between viable and non-viable target bacteria, the Ct values generated from PMA-treated live and dead cells after qPCR were compared to those of the corresponding PMA-untreated controls (Figure 1). For both microorganisms, treatment with 25 μM PMA concentration caused the lowest inhibition of qPCR from viable cells, as indicated by the minimum increase in the Ct value of DNA derived from PMA-treated live cells compared to the PMA-untreated controls (Figures 1A, B); and the highest inhibition from non-viable cells, as deduced by maximum increase in the Ct value of DNA from PMA-treated dead cells compared to the PMA-untreated controls (Figures 1C, D).

Hence, for both *L. acidophilus* and *B. bifidum*, the 25 μM PMA concentration allowed better detection of live cells with the lowest interference from dead cells.

**Figure 2:**
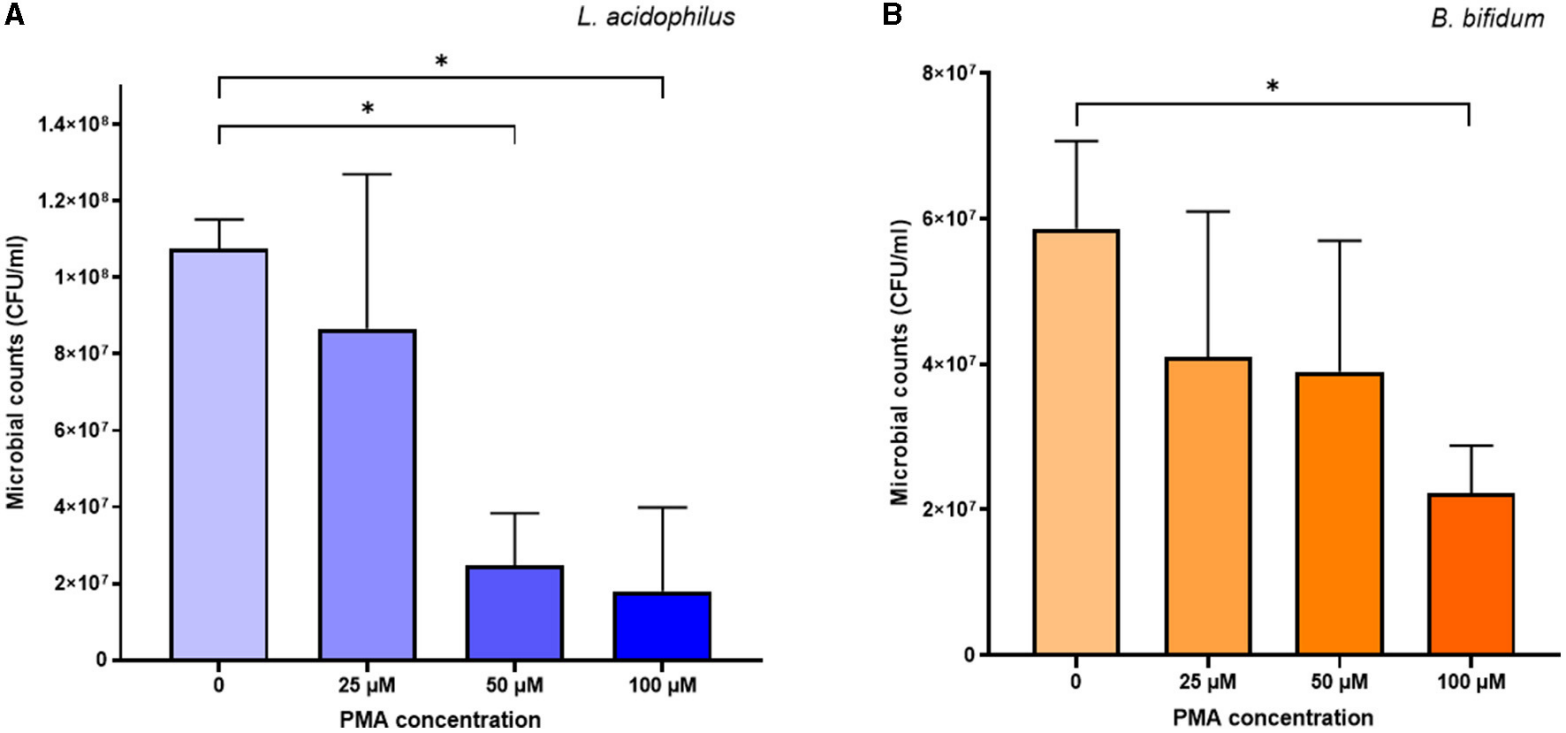
Cytotoxic effects of different PMA concentrations on *L. acidophilus* **(A)** and *B. bifidum* **(B)**. Bars depict the mean values of four experiments for each microorganism, with error bars representing the standard deviations. * *p* < 0.05.

Concerning the cytotoxic effects of PMA, the proportion of viable cells of both *L. acidophilus* and *B. bifidum* decreased as the PMA concentration increased (Figure 2). Compared to the PMA-untreated controls, the cytotoxic effects of PMA were significant at 50 μM (*p* = 0.0008) and 100 μM (*p* = 0.0026) for *L. acidophilus* (Figure 2A), and 100 μM for *B. bifidum* (*p* = 0.0101) (Figure 2B). The 25 μM PMA produced the least cytotoxic effects on both microorganisms, with no significant differences observed compared to the PMA-untreated samples.

Based on the above results, 25 μM PMA was selected to pretreat samples in the vPCR method.

### 3.4 qPCR of live and dead cells for verification of optimal PMA treatment

**Figure 3:**
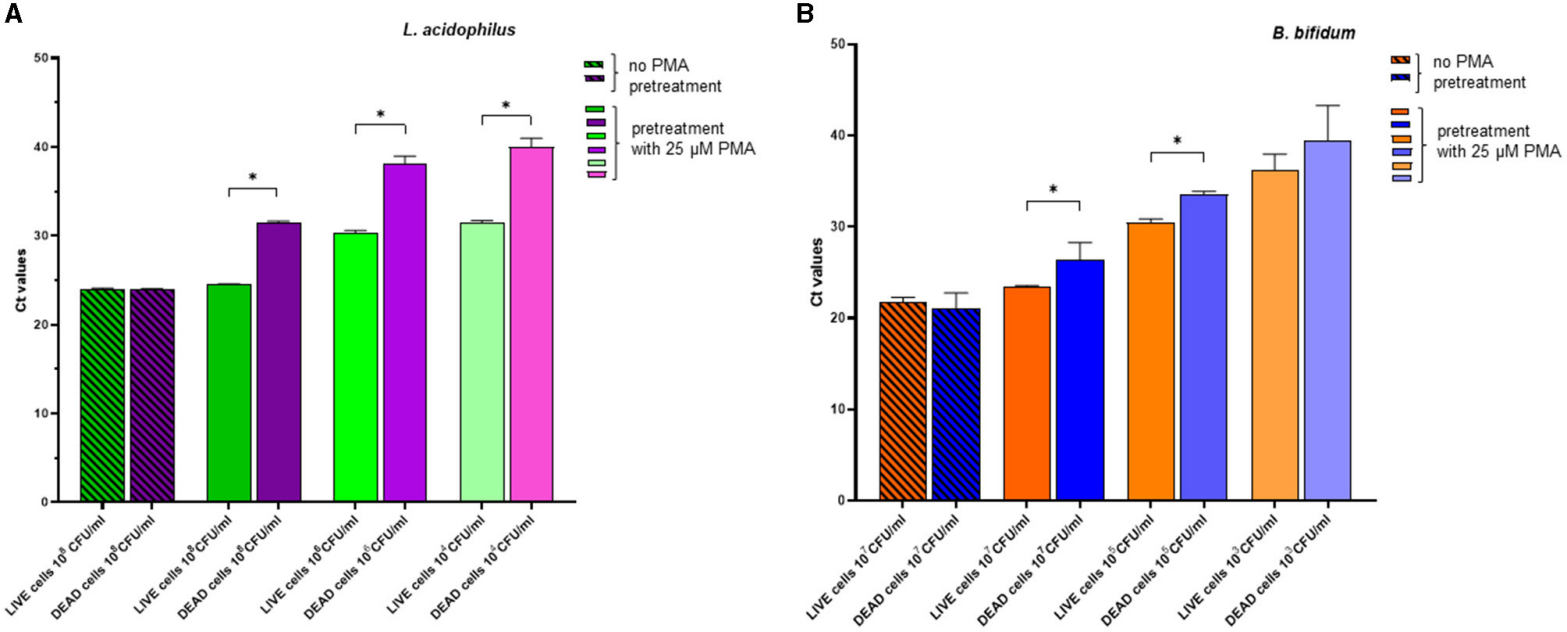
Effects of pretreatment with 25 μM PMA of different dilutions of live cells and dead cells of *L. acidophilus* **(A)** and *B. bifidum* **(B)** on the Ct values after qPCR. For each microorganism, the experiments were repeated twice with two replicates used in each experiment. Bars depict mean values with error bars representing the standard deviations. * *p* < 0.05.

Figure 3 shows the effects of pretreatment using the optimal PMA concentration (25 μM) on live and dead cells of *L. acidophilus* and *B. bifidum*, respectively. For both microorganisms, in the absence of PMA pretreatment, the Ct values of DNA derived from live and dead cells were comparable, indicating that DNA was amplified irrespective of cell viability. In contrast, PMA pretreatment resulted in a significant increase in the Ct values of DNA from dead bacteria compared with those from live bacteria (*p* < 0.0001 for *L. acidophilus* and *p* = 0.0210 for *B. bifidum*). The reduction in the qPCR signal indicated effectively inhibited DNA amplification from dead cells. This result was confirmed when the live and dead cells of both microorganisms were serially diluted (Figures 3A, B).

**Figure 4:**
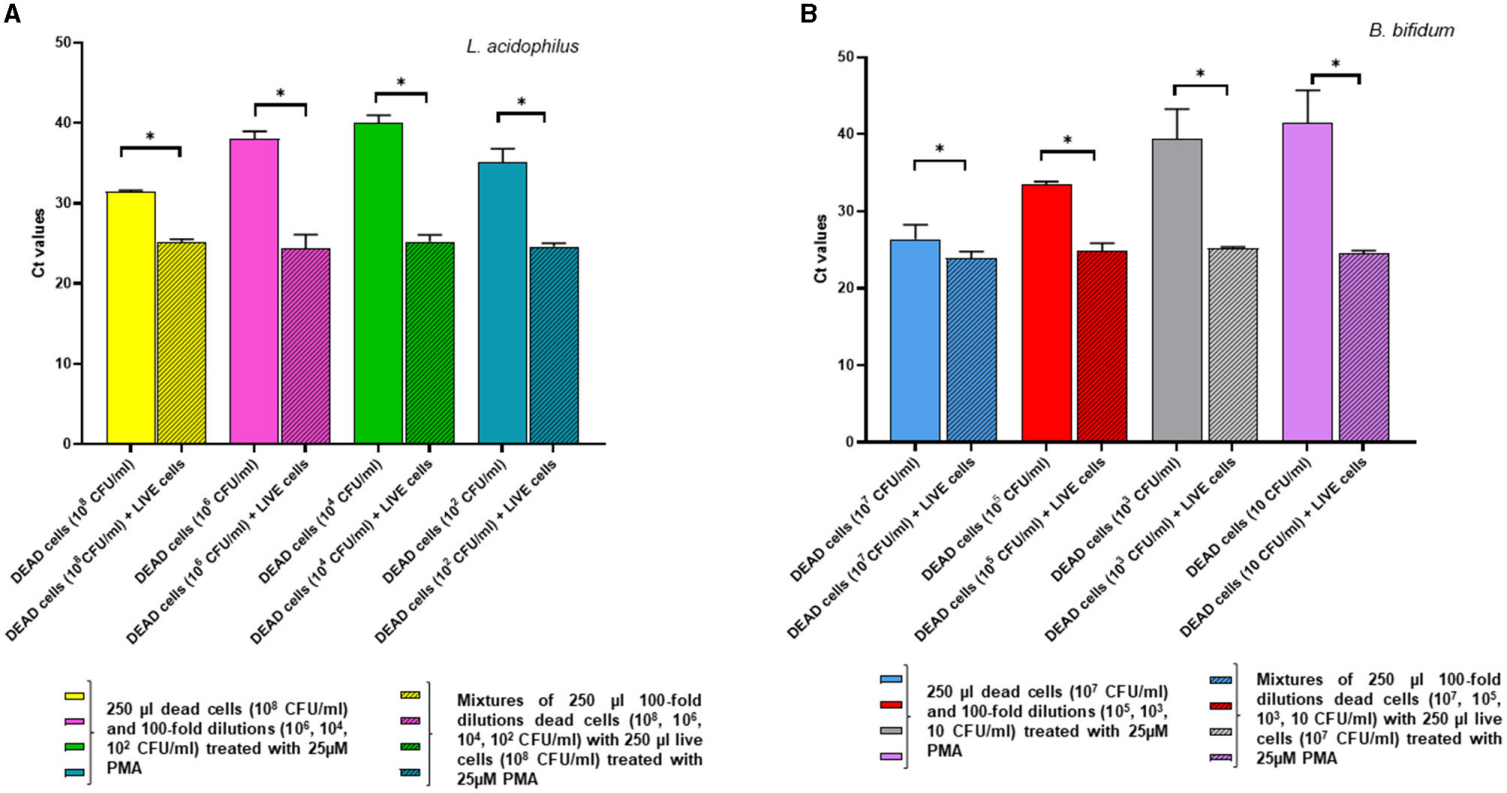
Effects of pretreatment with 25 uM PMA on qPCR of DNA derived from dead cells at decreasing concentrations, either alone or added with a fixed amount of live cells. Two replicates were used for each cell mixture and control, and each experiment was repeated twice. Bars depict mean values with error bars representing the standard deviations. **(A)** *L. acidophilus* and **(B)** *B. bifidum*. * *p* < 0.05.

In Figure 4, the effects of PMA pretreatment on qPCR of DNA derived from dead cells at decreasing concentrations, either alone or in combination with a fixed quantity of live cells, are shown for *L. acidophilus* and *B. bifidum*. For both microorganisms, the addition of live cells to different amounts of dead cells always produced significantly lower Ct values (*p* < 0.0001), as expected, because PMA should allow PCR amplification from live cells while suppressing amplification from dead cells. The fact that, for both microorganisms, the Ct values of all tested dead/live bacterial mixtures were very similar, regardless of the different dead cell concentrations, further demonstrates that DNA was essentially amplified from the live cells present in all mixtures in the same amount (Figures 4A, B).

**Figure 5:**
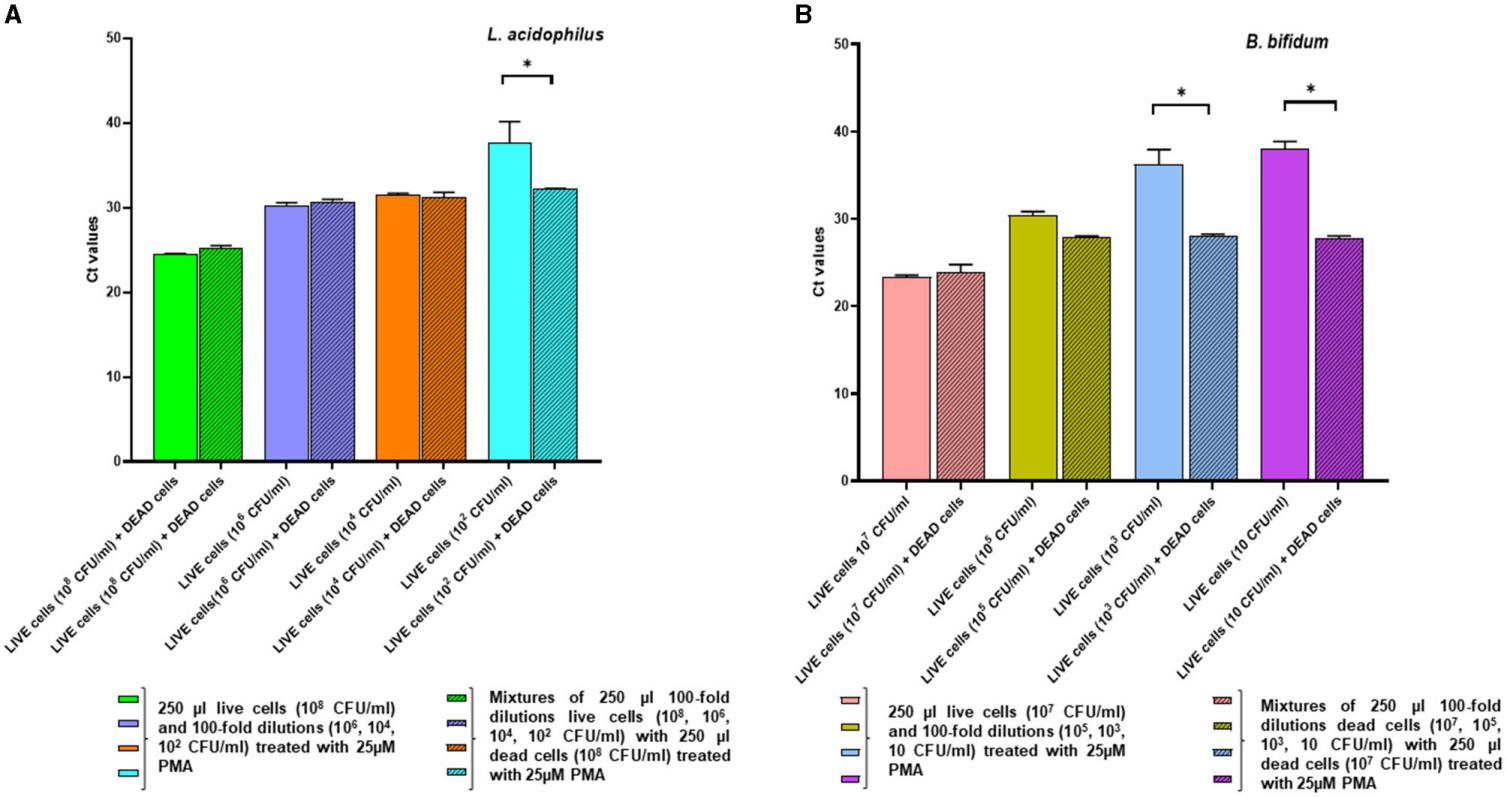
Effects of pretreatment with 25 uM PMA on qPCR of DNA derived from live cells at decreasing concentrations, either alone or added with a fixed amount of dead cells. Two replicates were used for each cell mixture and control, and each experiment was repeated twice. Bars depict mean values with error bars representing the standard deviations. **(A)** *L. acidophilus* and **(B)** *B. bifidum*. * *p* < 0.05.

In the complementary experiment, for *L. acidophilus* the addition of a fixed quantity of dead cells to decreasing numbers of live cells followed by PMA pretreatment did not affect the Ct values compared to those of the live cells alone, confirming that PMA efficiently inhibited qPCR amplification from the dead cells. However, inhibition of DNA amplification by PMA treatment was not evident at the lowest *L. acidophilus* live cell concentration tested at 10^2^ CFU/ml, as they generated a significantly higher Ct value compared to that of the relative mix with dead cells (*p* = 0.0055) (Figure 5A).

For *B. bifidum*, PMA inhibition of qPCR from dead cells was apparent when live cells in the live/dead cell mixtures were present at relatively high levels (i.e., 10^7^ CFU/ml and 10^5^ CFU/ml); in fact, at these concentrations there was no significant difference between the Ct values derived from live cells alone and the live/dead cell mixtures (Figure 5B). As for *L. acidophilus*, PCR inhibition by PMA pretreatment was not observable when the *B. bifidum* live cells quantities in the live/dead cell mixtures decreased ( ≤ 10^3^ CFU/ml), as demonstrated by the significant increase in the Ct values of the live cells alone compared to those of the relative mixtures with dead cells (*p* < 0.0001) (Figure 5B). This result could be due to the fact that, when the live cell concentration decreased in the mixtures containing high dead cell ratios, the 25 μM PMA pretreatment reduced the amplification from the viable cells while not fully inhibiting amplification from the dead cells, in accordance with what observed by other authors (Papanicolas et al., 2019).

Thus, our overall data indicate that the live cells quantification limits for the proposed vPCR assay, consisting of qPCR preceded by 25 μM PMA treatment, were approximately 10^2^ CFU/ml for *L. acidophilus* and 10^3^ CFU/ml for *B. bifidum*.

### 3.5 Application of the optimized vPCR protocol to the analysis of a commercial LBCP

**Figure 6:**
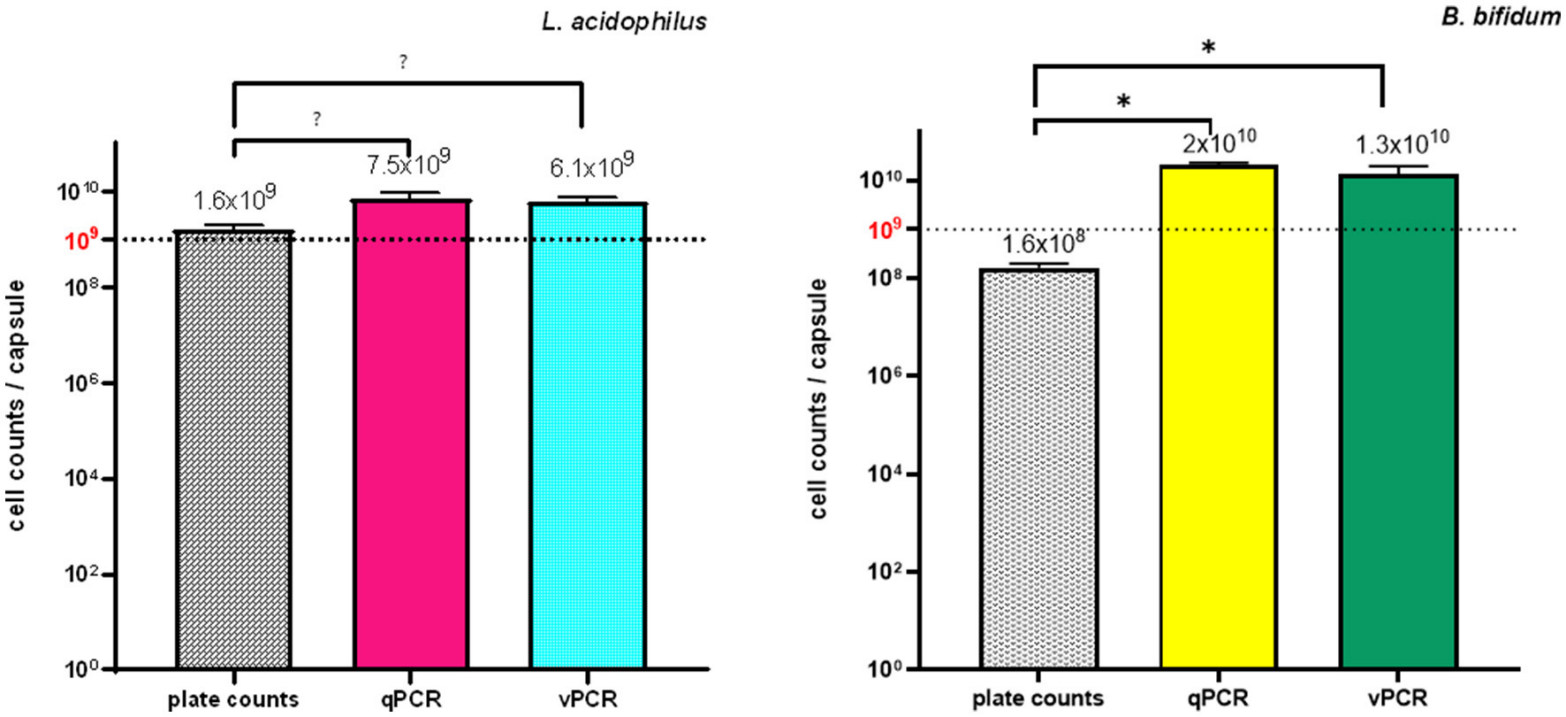
Quantities of *L. acidophilus* and *B. bifidum* in a commercial LBCP as determined by PC, qPCR and vPCR. Five LBCP capsules were analyzed. Each qPCR and vPCR reaction was performed in duplicate using *L. acidophilus* and *B. bifidum* specific primer/probe sets in separate reactions. Bars depict mean values with error bars representing the standard deviations. **(A)** *L. acidophilus* and **(B)** *B. bifidum*. * *p* < 0.05.

Figure 6 shows *L. acidophilus* and *B. bifidum* contents in a commercial LBCP, as determined by PC, qPCR, and the newly developed vPCR protocol.

Plate counts confirmed the presence of at least 10^9^ CFU/capsule of *L. acidophilus*, which was consistent with the declared labeled amount for that microorganism. For *B. bifidum*, whose stated label claim was also ≥10^9^ CFU/capsule, both counts on MRS agar supplemented with cysteine and on BSM agar plates yielded ~10^8^ CFU/capsule (Figure 6). Because *B. bifidum* is a “fastidious” microorganism to grow, being strictly anaerobic and nutrient-demanding, its concentration may have been underestimated using PC (Modesto, 2018). The production of inhibitory substances by *L. acidophilus* (the other microorganism present in the product formulation), such as organic acids and bacteriocins, or competition for nutrients on agar plates may also have contributed to the quantitative inconsistency between the product label and PC results for *B. bifidum*. Alternatively, it is possible that VBNC *B. bifidum* cells were present in the test samples and escaped culture detection.

The presence of VBNC microbial cells might also account for the significantly lower quantitative values obtained for both microorganisms using PC compared to those estimated using qPCR and vPCR (all *p* values < 0.001) (Figure 6): in fact, while VBNC microbial cells fail to grow in culture media, their DNA can be amplified using PCR.

The quantity estimates of both bacteria using vPCR were lower than those determined using qPCR, as expected, since amplification of DNA from dead cells in the samples should be prevented by the PMA pretreatment step of the vPCR protocol. However, the lack of a statistically significant difference between the quantitative values obtained using qPCR and vPCR for both microorganisms suggests that the tested LBCP contained a few dead cells (Figure 6).

## 4 Discussion

Qualitative and quantitative estimations of viable microorganisms deliberately added to LBCPs are essential to guarantee product efficacy and are required before marketing (FAO/WHO, 2002; Council for Responsible Nutrition International Probiotics Association, 2017; European Pharmacopoeia Commission, 2019).

The vPCR method described in this study allowed the identification and quantification of viable *L. acidophilus* and *B. bifidum* with adequate specificity, accuracy, and sensitivity of detection for testing LBCPs, which typically contain >10^6^-10^7^ CFU/g of live microorganisms to provide effective daily intake (Dinkçi et al., 2019; Marco et al., 2020; Boyte et al., 2023).

Once applied to the analysis of a LBCP containing both *L. acidophilus* and *B. bifidum*, the vPCR method showed better performance compared to both “gold standard” culture-dependent PC enumeration and the molecular approach of qPCR, which is also frequently used for routine microbiological testing purposes.

Although traditional PC enumeration relies on the ability of live microorganisms to multiply and form colonies on agar plates, vPCR uses membrane integrity as a viability criterion, thus including VBNC cells that are unable to grow on culture media (Davis, 2014; Bagheripoor-Fallah et al., 2015).

Indeed, our results from the LBCP analysis showed that the quantitative estimates of viable *L. acidophilus* and *B. bifidum* using vPCR were significantly higher than colony counts, suggesting that VBNC cells of both microorganisms were present in the product, likely because bacteria can easily enter the VBNC state in response to the manufacturing process (Oliver, 2005; Kumar and Ghosh, 2019).

Notably, VBNC cells in LBCPs can still exert beneficial effects on the host (Adams, 2010), can be resuscitated, depending on environmental factors, and restore full metabolic activity and the ability to multiply (Oliver, 2005; Kumar and Ghosh, 2019). Therefore, detecting VBNC microbial cells is not only essential for pathogens because of the risk that they can regain virulence upon resuscitation, but is equally significant for obtaining a more reliable quantification of the total viable beneficial bacteria in a product.

Our study confirms that, being able to detect VBNC cells, vPCR can provide more accurate quantitative estimates of viable microorganisms in a sample compared to classic microbiological culture-based methods and in a shorter time, considering the relatively long incubation periods required for bacterial cultivation. In addition, the advantage of detecting VBNC cells outweighs any disadvantages caused by the higher economic costs and sophisticated systems required to perform vPCR vs. PC enumeration. The potential applicability of the method to the detection of multiple microorganisms in a single test might reduce the overall costs if large numbers of samples per day are to be analyzed, as in routine control screening.

Compared with qPCR, which cannot distinguish between DNA from live and dead microbial cells, vPCR enables the selective detection of viable microbial cells (Kumar and Ghosh, 2019; Boyte et al., 2023). Accordingly, our results of the LBCP analysis using the vPCR method showed lower quantitative amounts of both tested microorganisms than those estimated using qPCR.

In conclusion, the vPCR assay proposed here allowed for the accurate identification, quantification, and viability determination of both *L. acidophilus* and *B. bifidum* in approximately 5 h, thus representing a reliable high-throughput molecular test for the microbiological quality assessment of LBCPs containing these microorganisms.

A potential limitation of this method is that it is species specific rather than strain specific, whereas the potential health benefits of the microorganisms to be included in LBCPs, as well as any potential concerns, should be demonstrated at the strain level (European Pharmacopoeia Commission, 2019; EFSA Panel on Biological Hazards, 2023). Nevertheless, although a consensus definition of microbial strain based on more recent genomic knowledge is still needed, species-specific methods may be considered acceptable for the analysis of products that do not contain individual strains of the same species (Boyte et al., 2023), currently representing the majority of LBCPs available on the market. Availability of the whole genome sequences from strains used in LBCPs would be necessary in order to be able to detect and quantify viable bacteria at the strain level by a molecular method.

## Data availability statement

The raw data supporting the conclusions of this article will be made available by the authors, without undue reservation.

## Author contributions

SC: Data curation, Formal analysis, Supervision, Writing – review & editing. SI: Data curation, Formal analysis, Investigation, Writing – review & editing. DG: Conceptualization, Formal analysis, Methodology, Supervision, Writing – review & editing. CVH: Writing – review & editing. GF: Conceptualization, Methodology, Supervision, Writing – original draft, Formal analysis.
